# Study of the hearing in children born from pregnant women exposed to occupational noise: Assessment by distortion product otoacoustic emissions

**DOI:** 10.1016/S1808-8694(15)30080-X

**Published:** 2015-10-19

**Authors:** Eduardo Bezerra Rocha, Marisa Frasson de Azevedo, João Aragão Ximenes Filho

**Affiliations:** a MD. Otorhinolaryngologist - SBORL; M.S in Human Communication - UNIFESP-EPM/ UNIFOR. Professor; b PhD in Human Communication - UNIFESP, Adjunct Professor and Vice-chairperson of the Hearing Disorders Department - Speech and Hearing Therapy - EPM; c PhD in Otorhinolaryngology - FMUSP, Guest Postgraduate Program Professor of Surgery - FMUFC

**Keywords:** hearing, occupational/prevention, control, noise

## Abstract

A**im:** To detect early on a probable hearing loss in children of women exposed to occupational noise during their pregnancy and to verify if there is any difference between the children from those women exposed to occupational noise during their pregnancy and the ones from mothers that do not work under the same conditions. **Methods:** Children from women exposed to occupational noise during their pregnancy and children from women who were not exposed were evaluated through distortion product otoacoustic emissions, using the GSI 60 DPOEA SYSTEM equipment and the frequency-ratio F_2_/F_1_ equal to 1.2 and the geometric average of 2F_1_-F_2_. The intensity of the primary frequencies were kept steady with values of L1=65dBSPL and L2=55dBSPL for F_1_ and F_2_, respectively. Student T test in paired samples and independent samples were used. **Results:** There were no differences in the response amplitude of distortion product otoacoustic emissions between the control and the study groups. There was no statistically difference between male and female children in response amplitude for the two groups aforementioned; and there were no differences between right and left ears from each group. **Conclusion:** We did not observe hearing impairment in children whose mothers were exposed to occupational noise during pregnancy when compared to the children from mothers who were not. There was no difference between the right and left ears, nor between male and female children in each group.

## INTRODUCTION

Although temporary, sound pollution is responsible for great harm. Such pollution, dealt upon as noise, can be social or occupational. Social noise is the one caused by discos, noisy cars, rock bands, usually of short duration and that, if isolated and episodic, would hardly cause hearing problems to men^1^.

In general, those who work exposed to noise levels above 85dBSPL and under long standing exposure must be monitored. Auditory monitoring can be carried out by means of threshold tonal audiometry, which determines the least sound intensity capable of causing auditory sensation in each frequency tested, using a pure sound stimulus, and this is the one most employed in factories.

Another method is evoked otoacoustic emmission2-9 which is obtained in response to previous sound stimulation, and it can be with pure tones - by distortion products of pre-established frequencies, which analyzes cochlear activities in specific frequencies^10^, ^11^, ^12^, ^13^ or by a very short sound stimulus, a “click” - transitory and that represent a global cochlear response^11^. It is an objective, quick, non-invasive and easily applicable method 14, 15, 16, 17, including the screening of children in high risk nurseries^18^.

Until very recently, the concern in assessing the hearing of those exposed to noise was limited to males. However, with the need to contribute financially to the household, women today represent almost 50% of the work force and are present in a sizeable share of the world industrial setting and, most of them are in their reproductive years^19^. It is known that there is a greater individual susceptibility; however, there is no clinical evidence that men or women be more prone to hearing problems when exposed to noise.

It is known that when a sound wave goes from the air medium (outer and middle ear) to the liquid medium (labyrinthine liquids), 99.9% of this energy is lost. It is also known that the human fetus is protected inside the mother’s womb and the tissues and liquid around this harmless being may damp environmental noise^20^, ^21^ and, according to a quote from Niemtzow^22^ (1982), such damping shall be more effective as the frequency increases. Thus, lower frequencies are less damped, and therefore are the most harmful to the fetal cochlea.

Many are the papers that discuss the effects of occupational noise on the fetus^21^, ^22^, ^23^, ^24^, ^25^, ^26^; however, few approach its effects on the fetus’s hearing27. It is debated whether or not noise may cause low birth weight^28^, ^29^ or bring about alterations to the immune system30. The possible devastating effect occupational noise may have on this future human has been considered, and thus we deemed relevant to invest in this research project, trying to establish a cause/effect relation in regards of these women and their work environment.

In the present investigation, the target public is, therefore, the children born from women who, during pregnancy were exposed to occupational noise. Since tonal audiometry is a method that is impossible to be used in neonates because it requires a behavioral answer from the examinee, otoacoustic emissions bear the ideal traits to be the exam of choice in these cases^3^, ^12^.

The goals of the present investigation were:

1. Provide for an early detection of hearing loss in children born from women exposed to occupational noise during pregnancy;
2. Check to see whether there is any difference in the results of otoacoustic emissions - distortion product - amplitudes among the children born from mothers who were exposed to occupational noise and the children born from mothers who were not exposed to occupational noise.

## PATIENTS AND METHODS

### Patients

This work was developed at the department of Speech and Hearing Therapy of the Núcleo de Atenção Médica Integrada (NAMI), of the University of Fortaleza (UNIFOR), from August 2002 through June of 2003. Patients underwent otorhinolaryngology exam, followed by otoacoustic emissions - distortion product (DPOAM), after informing the guardian and obtaining a signed consent. The study was approved by the Ethics Committee of both universities: University of Fortaleza and the Federal University of São Paulo (UNIFESP-EPM), protocol # 0330/03.

The object of our study were children with ages ranging from 0 to 6 months, of both genders and without any risk factor for hearing loss, according to the recommendations established by the preliminary screening group of neonatal screening, formed by the Brazilian Society of Otorhinolaryngology in May of 2000. Those children who presented some risk indicator for hearing loss were taken off the study.

The women (mothers) come from the same socio-economical background (family income equivalent to 1-2 minimum wages). We also excluded those women who worked exposed to chemical products and who smoked during pregnancy^26^.

Questionnaires were answered during interview with the researcher on the pregnancy and delivery conditions, considering birth weight and height, mother’s and relatives’ past, and that of the child being investigated, and also the mother’s professional activity during pregnancy. Thus, all the aforementioned variables were eliminated and the children were selected.

All the children were submitted to an otorhinolaryngological evaluation, in an attempt to investigate cranio-facial malformations and rule out external and/or middle ear involvement, by means of an otoscopy. Those children with external and/or middle ear problems were treated clinically and then reassessed. When there were within normal parameters, they were approved to join the study.

The study was carried out with 80 children who were distributed in two groups: study and control. The first was made up of 35 children with ages varying between 0 and 6 months, of both genders, born from women exposed to occupational noise above 80dBSPL (intensity range above 80dBSPL and below 90dBSPL), in an 8 hour daily work routine, making up a total of 40 hours per week of work during pregnancy, and whom both the gestation and delivery happened without complications. The children hereby studied are from women who worked in a nuts processing plant, located in the city of Cascavel, in the state of Ceará, 60 Km away from Fortaleza. All these women worn personal protection equipment (PPE) - ear plug - during their work routine and kept working exposed to occupational noise until at least the 8th month of pregnancy, and most took maternity leave 15 days before delivery (Table 1).

**Table 1:** Children distribution by gender and group.

	Males	Females	Total
Control Group.	26 (57.7%)	19 (42.3%)	45
Study Group.	16 (45.7%)	19 (54.3%)	35

The control group was formed by 45 children, also from both genders, with matching ages to those in the study, born from women who were not exposed to occupational noise during gestation, from the Dendê community and who are followed at the NAMI, without the same hearing loss risk factors of those children in the study, who went through the same assessment procedure (Table 1).

### Method

All women in both groups received prenatal care and conceived children in public maternities under the care of a health care professional. Data on birth conditions and the child’s status at delivery were collected from the child’s vaccination card or from the birth records provided by the maternity.

### Otoscopic Exam

The equipment used for the otorhinolaryngological assessment were a Kole headlamp and a Heine otoscope. This assessment was necessary in order to check external and middle ear integrity, which function is essential in order to properly capture the otoacoustic emissions.

### Distortion Product - Evoked Otoacoustic Emissions

The children who passed the otorhinolaryngological exam were submitted to auditory analysis by means of the distortion product - evoked otoacoustic emissions.

In order to record the DPOAE we used a GSI 60 DPOEA SYSTEM. Recording took place after the generation of two pure tones F1 and F2, where F2 was always higher than F1 and were called primary frequencies. F2 varied between 593 Hz and 6031 Hz and its geometric average (GA) followed the 2F1-F2 standard in the F2/F1 ratio equal to 1.2. The primaries F1 and F2 (L1 for F1 and L2 for F2) stimuli intensities remained fixed at 65dBSPL and 55dBSPL, respectively, in other words, L1 was greater than L2 in 10dBSPL. The DPOAE measures were carried out from the low to the high frequencies.

An acoustic probe with two receivers (micro-speakers) was coupled to the measuring device, which were responsible for the emission of acoustic signals (F1 and F2) and 1 high sensitivity miniature microphone used to collect the DPOAE, and this whole set of transducers were assembled in a small probe similar to the one used in immitanciometry, and which was coupled to the external auditory meatus by means of a rubber plug of proper size for each child tested.

The device was calibrated whenever turned on, using a 2cm^3^ cavity simulator, where we checked the correct sealing and working of the transducers and necessary adjustments for calibration purposes were made by the device itself.

The distortion products and background noises were recorded and analyzed in relation to the sound frequencies, as shown on Table 2.

**Table 2:** F_1_ and F_2_ primary frequencies and distortion product for 2 F_1_-F_2_

Primary					
Point	F_1_	F_2_	F_2_/F_1_	GM(Hz)	DP 2F_1_-F_2_(Hz)
1	500	593	1,2	531	406
2	625	750	1,2	687	500
3	781	937	1,2	843	625
4	1000	1187	1,2	1093	812
5	1250	1500	1,2	1375	1000
6	1593	1906	1,2	1750	1281
7	2000	2406	1,2	2187	1593
8	2531	3031	1,2	2781	2031
9	3187	3812	1,2	3500	2562
10	4000	4812	1,2	4375	3187
11	5031	6031	1,2	5500	4031

DPOAE were considered present whenever the distortion product amplitude value were positive, with a difference equal to, or higher than 6dBSPL in relation to the background noise, in other words, a signal to noise ration difference equal to or higher than 6dBSPL. Negative amplitudes, with differences below 6dBSPL were considered absent. We always tried to keep noise levels below zero (negative). Cases of excessive noise, masking the response, were not analyzed.

The exam was carried out in an acoustically treated room and booth, in the selected children that, after fed, were sleepy or slightly asleep and in the arms of the mother or guardian.

### Statistical analysis

The statistics used for data analysis was based on the t-Student test for averages comparison in: (i) paired samples (dependent) and (ii) independent samples.

The significance level used in the tests conclusion was of 0.05 in all the tables and we present edthe descriptive level of the tests (p-value), in other words, minimum significance level to be used for ruling out the hypothesis H0. Data were analyzed by means of the SPSS version 8.0 statistics software.

## RESULTS

Study of the distortion product amplitudes and background noise in function of the ear side variable in both the control and the study groups

Hereby we present the results from the distortion product - evoked otoacoustic emissions and back ground noise in children born from mothers who were not exposed to occupational noise during pregnancy (control group), analyzing their response amplitudes for each ear and, following that, comparing them among each other. We used the t-Student test for paired samples (significant for p < 0.05) and there was no statistically significant difference between right and left ears for the distortion product and background noise amplitude averages in the control group. As for the study group, the right ear presented a statistically significant difference in the frequency of 1500 Hz when compared to the left ear, in other words, the right ear presented better distortion product amplitude average when compared to the left ear. As for background noise, there was no statistically significant difference between right and left ears in the study group.

**Table 3:** mean values, standard deviation and student t test (paired samples) in order to compare DP (distortion product) mean values obtained for right and left ears – control group

F2 Hz	Right Ear		Left Ear		t	gl	p-value
	DP Mean value	Standard Deviation	DP Mean value	Standard Deviation			
593	9,24	7,69	12,16	8,73	-1,617	44	,113
750	3,51	7,01	5,33	9,18	-1,178	44	,245
937	6,82	6,19	7,49	6,98	-0,645	44	,523
1187	10,73	6,39	9,62	7,06	1,189	44	,241
1500	12,60	7,94	12,93	6,81	-,321	44	,750
1906	15,62	7,09	14,73	6,52	1,019	44	,314
2406	14,51	5,20	14,44	5,46	,083	44	,934
3031	10,49	4,75	9,98	4,92	,737	44	,465
3812	10,93	5,78	9,98	5,30	1,144	44	,259
4812	8,20	5,96	6,47	5,70	1,779	44	,082
6031	4,58	4,21	3,20	4,99	1,881	44	,067

**Table 4:** mean values, standard deviation and student t test (paired samples) in order to compare BN (background noise) mean values obtained for right and left ears – control group

F2 Hz	Right Ear		Left Ear		t	gl	p-value
	Mean BN	Standard Deviation	Mean BN	Standard Deviation			
593	8,78	8,36	10,80	7,18	,618	44	,540
750	4,27	6,67	3,76	7,79	,808	44	,424
937	0,89	4,87	2,07	5,41	-1,350	44	,184
1187	-0,20	4,86	0,80	5,85	,369	44	,714
1500	-3,20	5,28	-1,91	5,74	-1,154	44	,255
1906	-5,73	4,01	-4,31	4,13	-,890	44	,378
2406	-8,16	2,77	-7,44	3,66	-1,172	44	,248
3031	-9,82	4,26	-8,84	3,58	-1,510	44	,138
3812	-9,73	3,36	-9,09	2,87	-,961	44	,342
4812	-8,27	2,78	-8,60	2,13	-1,211	44	,232
6031	-7,31	2,08	-7,62	2,22	-1,091	44	,281

**Table 5:** mean values and standard deviation of the distortion product difference with the background noise (a=DP-BN) obtained for the right and left ears – control group

F2 Hz	Right Ear		Left Ear	
	Mean A	Standard Deviation	Mean A	Standard Deviation
593	0,47	6,25	1,27	6,45
750	-0,76	6,31	1,58	7,37
937	5,93	7,30	5,42	7,39
1187	10,89	8,18	8,73	8,05
1500	15,80	8,65	14,84	8,14
1906	21,36	9,30	19,04	7,44
2406	22,67	5,87	21,89	5,78
3031	20,31	6,57	18,82	5,87
3812	20,67	5,68	19,07	5,80
4812	16,27	5,42	15,09	4,94
6031	11,89	3,08	10,82	3,87

**Table 6:** mean values, standard deviation and student t test (paired samples) in order to compare DP (distortion product) mean values obtained for right and left ears – study group

F2 Hz	Right Ear		Left Ear		t	gl	p-value
	Mean value DP	Standard Deviation	Mean value DP	Standard Deviation			
593	9,46	11,10	11,71	8,31	-,966	34	,341
750	5,00	6,04	5,29	6,73	-,233	34	,817
937	7,80	6,41	7,51	6,36	,210	34	,835
1187	10,51	5,52	8,37	7,73	1,653	34	,108
1500	15,03	5,46	13,00	7,19	2,396	34	,022 (*)
1906	15,34	5,78	14,09	6,99	1,204	34	,237
2406	15,49	6,34	14,34	5,70	1,145	34	,260
3031	10,46	4,84	9,23	4,53	1,577	34	,124
3812	10,77	5,92	10,34	4,29	,415	34	,680
4812	8,49	5,87	7,31	4,23	1,039	34	,306
6031	4,89	5,97	2,80	5,66	1,646	34	,109

Note: (*) Significant at 5%

**Table 8:** mean values and standard deviation of the distortion product difference with the background noise (a=DP-BN) obtained for the right and left ears – study group

F2 Hz	Right Ear		Left Ear	
	Mean A	Standard Deviation	Mean A	Standard Deviation
750	3,26	7,32	2,66	7,62
937	7,63	7,38	7,00	7,19
1187	12,74	6,73	10,77	9,48
1500	18,11	7,34	15,94	8,11
1906	20,60	7,92	19,66	6,23
2406	23,31	6,90	22,23	6,86
3031	20,74	6,74	18,43	5,94
3812	19,91	5,56	19,66	4,99
4812	17,11	5,36	15,57	4,14
6031	12,23	4,99	10,94	4,49

Study of the distortion product amplitudes and background noise in function of the gender variable in both the control and the study groups

We analyzed the average with standard deviation measures for distortion product - evoked otoacoustic emissions and background noise in relation to gender for each group (study and control).

Boys presented better distortion product average amplitudes when compared to girls in the F2 frequencies of 1187 KHz and 1500 KHz for the control group, while girls had better distortion product amplitude averages when compared to boys in the F2 frequency of 2406 KHz. There was no statistically significant difference for background noise insofar as gender is concerned for both groups.

Comparative study of background noise and distortion product amplitudes between the control and study groups

Considering both ears together for the assessed group. There was no statistically significant difference between the two groups in relation to the averages of response amplitudes in distortion product. Both for the control and the study groups, background noise presented positive values for the low frequencies and negative values for the (F2) frequencies starting at 1500Hz for the control group and at 1187Hz for the study group.

**Table 11:** mean values, standard deviation and t-student test (independent samples) in order to compare DP mean values assessed by gender – study group

F2 Hz	Male		Female		p-value ^1^ F	p-value ^2^ t
	Mean value DP	Standard Deviation	Mean value DP	Standard Deviation		
593	11,6563	10,5788	9,6842	9,1359	,244	,406
750	5,6563	6,2919	4,7105	6,4511	,529	,539
937	7,6875	5,0189	7,6316	7,3427	,037	,971
1187	9,6875	7,5024	9,2368	6,1444	,549	,783
1500	14,6563	4,7831	13,4737	7,5507	,068	,447
1906	13,7500	6,7633	15,5263	6,0437	,171	,250
2406	13,2813	6,1184	16,2895	5,6325	,862	,036 (*)
3031	9,0313	4,2539	10,5263	4,9850	,267	,186
3812	10,4688	4,1034	10,6316	5,9248	,232	,896
4812	7,2813	4,6851	8,4211	5,4556	,410	,357
6031	4,0938	5,7774	3,6316	6,0154	,972	,745

**Note:** (1) Levene test for equality of variances, (2) t-Student test for independent samples

(*) Significant at 5%

**Table 12:** mean values, standard deviation and t-student test (independent samples) in order to compare BN mean values assessed by gender – study group

F2 Hz	Male		Female		p-value ^1^ F	p-value ^2^ t
	Mean value DP	Standard Deviation	Mean value DP	Standard Deviation		
593	10,1875	8,0380	6,7105	9,1087	,177	,098
750	3,4688	6,5401	,2105	8,1046	,241	,072
937	1,2188	5,0910	-,3947	6,0159	,154	,235
1187	-2,2188	5,1852	-2,3947	5,4204	,918	,891
1500	-3,4688	4,9186	-2,7368	5,6792	,392	,570
1906	-5,9063	3,9132	-4,7632	4,8123	,115	,285
2406	-7,7813	2,5870	-8,1316	3,6993	,151	,654
3031	-9,2188	3,3672	-10,1316	3,8985	,247	,303
3812	-9,0625	2,9175	-9,3684	3,2832	,581	,684
4812	-8,3750	2,4330	-8,5000	2,9201	,961	,848
6031	-7,5625	2,9614	-7,8947	2,5126	,525	,613

**Note:** (1) Levene test for variances analyses, (2) t-Student test for independent samples.

## DISCUSSION

The choice for distortion product - evoked otoacoustic emissions was based on their wide clinical application:

1) in assessing the cochlear external hair cells function^11^;
2) it is frequency-specific;
3) recommended for use in babies and
4) are able to detect changes in auditory threshold even before conventional tonal audiometry does^9^.

In order to obtain a better response amplitude and less background noise interference, distortion productevoked otoacoustic emissions were applied to the population studied, following the criteria: F2/F1 equal to 1.2, geometric average of the F1 and F2 primary frequencies in 2F1-F2; and the stimuli intensities of F1 and F2 were of 65 dB SPL and 55 dB SPL (that is: L1=L2-10), respectively^4^.

The distortion products-response-amplitude for both the right and left ears in the control group varied according to frequency F2, always presenting positive values in all the frequencies tested, the highest values were seen in the frequencies of 1500 Hz, 1906 Hz, 2406 Hz, 3031 Hz and 3812 Hz for both ears. The highest peak of response amplitude was seen at 1906 Hz and 2406 Hz, with values of 15.62 dBSPL and 14.51 dBSPL for the right ear, and values of 14.73 dBSPL and 14.44 dBSPL for the left ear in the same frequencies, respectively.

Authors such as Bonfils et al.^14^ (1993) and Abdala^12^ (1996) also found distortion product average amplitude responses in normal children with positive values in all the F2 frequencies tested.

As to the maximum response amplitude peaks, the literature shows values of 17.8 dBSPL in 2 KHz, of 16 dBSPL in 1.5 KHz12, of 17.26 dBSPL in 2 KHz, of 16.8 dBSPL in 1.5 KHz and of 17.4 dBSPL in 2 KHz. The results of the present investigation, in relation to the maximum distortion product amplitude response, are in agreement with those found in the literature; in other words, in the F2 frequency ranged around 2 KHz.

It was also seen that background noise average measures for both ears in the control group presented positive and high values in the lower frequencies and decreased as the frequencies become higher, taking on negative values starting in the frequency of 1187 Hz for the right ear and 1500 Hz for the left ear. The highest peak for negative values for background noise was seen in the frequencies of 3031 Hz and 3812 Hz; with values of -9.82 dBSPL and -9.73 dBSPL for the right ear; and values of -8.84 dBSPL and -9.09 dBSPL for the left ear.

Results from the present investigation agree with those who found background noise taking on higher values in the lower frequencies. Bonfils et al.^14^ (1993) reported that the background noise had higher values in the lower frequencies and lower values in the higher frequencies. The same thing was noticed in the present study.

In regards to the difficulty in recording distortion product responses in the F2 frequencies, in the present investigation we observed that below the F2 frequency of 1500 Hz, despite positive values for response average amplitudes, background noise was high and hampered response interpretation.

In relation to average amplitudes, given by the distortion product (signal) response amplitude difference with the background noise amplitude (table 7), it was observed that values equal to or above 6dBSPL were present starting in the F2 frequency equal to or higher than 1187 Hz for the right and left ears, showing maximum peaks in 1906 Hz and 2406 Hz with values of 21.36 dBSPL and 22.67 dBSPL for the right ear and in 2406 Hz and 3812 Hz with values of 21.89 dBSPL and 19.07 dBSPL for the left ear, respectively.

**Table 7:** mean values, standard deviation and student t test (paired samples) in order to compare BN (background noise) mean values obtained for right and left ears – study group

F2 Hz	Right Ear		Left Ear		t	gl	p-value
	Mean value DP	Standard Deviation	Mean value DP	Standard Deviation			
593	8,03	8,77	8,57	8,85	-,313	34	,756
750	1,74	8,06	1,66	7,14	,072	34	,943
937	0,17	6,37	0,51	4,87	-,311	34	,757
1187	-2,23	4,83	-2,40	5,76	,157	34	,876
1500	-3,09	5,17	-3,06	5,54	-,036	34	,971
1906	-5,26	4,41	-5,31	4,51	,061	34	,952
2406	-7,83	3,23	-8,11	3,25	,372	34	,712
3031	-10,23	4,02	-9,20	3,25	-1,298	34	,203
3812	-9,14	2,98	-9,31	3,26	,214	34	,832
4812	-8,63	3,12	-8,26	2,21	-,549	34	,586
6031	-7,34	2,03	-8,14	3,24	1,360	34	,183

We may see that the best signal/noise difference amplitude averages are obtained in the frequency range of F2 where the best distortion product response amplitudes are present, coinciding with the lower values for background noise.

When we applied the t-Student (paired samples) test to compare the distortion product amplitude responses and those of the background noise for the right and left ears, we did not observe statistically significant differences (p < 0.05) in any of the F2 frequencies tested.

In the present investigation, the distortion product amplitude averages for both, the right and left ears of the study group varied according to F2 frequency, presenting positive values in all tested frequencies of 1187 Hz, 1500 Hz, 1906 Hz, 2406 Hz, 3031 Hz and 3812 Hz of the right ear and in the frequencies of 593 Hz, 1500 Hz, 1906 Hz, 2406 Hz and 3812 Hz of the left ear. A maximum response amplitude peak was seen at 1906 Hz and 2406 Hz with values of 15.03 dBSPL and 15.34 dBSPL for the right ear, and values of 14.09 dBSPL and 14.34 dBSPL for the left ear, respectively.

By using the t-Student (paired samples) test in order to compare the distortion product amplitude response averages evaluated for the left and right ears of the study group, there was a statistically significant difference between the ears only in the F2 frequency of 1500 Hz, and the right ear presented better response amplitude averages when compared to the left ear. As seen above, there are controversies in relation to the ear side that presents the best response amplitudes, in other words, authors said they found better response amplitudes for the right ears, while others observed response amplitude averages similar for both the right and left ears. In the present investigation, only one F2 frequency (1500 Hz) presented response amplitude averages that were better for the right ear when compared to the left ear in the study group.

We observed that the background noise measures for both the right and left ears of the study group presented positive values for the lower frequencies, with high values in the F2 frequency of 593 Hz. It was also observed that the background noise decreased as the frequencies became higher, taking on negative values starting from F2 frequency of 1187 Hz for both ears (Table 9). Maximum peak of negative values for background noise occurred in the frequencies of 3031 Hz and 3812 Hz, with values of -10.23 dBSPL and -9.14 dBSPL, for the right ear, and with values of -9.20 dBSPL and -9.31 dBSPL, for the left ear.

**Table 9:** mean values, standard deviation and student t test (independent samples) in order to compare DP (distortion product) mean values assessed by gender – control group

F2 Hz	Male		Female		p-value ^1^ F	p-value ^2^ t
	Mean value DP	Standard Deviation	Mean value DP	Standard Deviation		
593	9,7692	7,7576	11,9737	8,9608	,582	,216
750	5,1154	8,6402	3,4737	7,5004	,807	,350
937	7,5192	6,5334	6,6579	6,6790	,915	,542
1187	11,4423	6,1911	8,4474	7,0966	,743	,036 (*)
1500	14,4615	6,3167	10,4474	8,1030	,251	,010 (*)
1906	16,0000	6,2151	14,0526	7,4360	,117	,180
2406	14,5962	4,6579	14,3158	6,1385	,088	,806
3031	10,0192	4,0752	10,5263	5,7127	,075	,624
3812	9,9231	5,0014	11,1842	6,1857	,138	,288
4812	6,9423	5,5850	7,8684	6,2522	,333	,462
6031	3,3462	4,9304	4,6316	4,1682	,153	,196

**Note:** (1) Levene test for equality of variances, (2) t-Student test for independent samples.

When we applied the t-Student (paired samples) test to compare background noises assessed for right and left ears, we did not find statistically significant differences between the ears, corroborating some aforementioned authors.

The amplitude averages of the signal/noise difference in the study group for the right and left ears presented values equal to or superior than 6 dBSPL from the F2 frequency of 937 Hz, showing maximum peaks of 20.60 dBSPL; 23.21 dBSPL and 20.74 dBSPL in the frequencies of 1906 Hz, 2406 Hz and 3031 Hz, for the right ear; and of 19.66 dBSPL; 22.23 dBSPL and 19.66 dBSPL in the frequencies of 1906 Hz, 2406 Hz and 3812 Hz, for the left ear, respectively (Table 10).

**Table 10:** mean values, standard deviation and t-student test (independent samples) in order to compare BN assessed by gender – control group

F2 Hz	Male		Female		p-value ^1^ F	p-value ^2^ t
	Mean value BN	Standard Deviation	Mean value BN	Standard Deviation		
593	9,3846	7,0909	10,3421	8,7774	,021	,582
750	4,0962	7,3518	3,8947	7,1236	,886	,897
937	1,9231	4,7023	0,8684	5,7147	,096	,340
1187	0,1346	5,7804	0,5263	4,8253	,367	,735
1500	-2,1923	5,7770	-3,0526	5,1933	,742	,469
1906	-4,9038	4,4336	-5,1842	3,6676	,289	,751
2406	-8,0769	2,9362	-7,4211	3,6364	,981	,347
3031	-9,0577	3,8573	-9,7105	4,0797	,775	,441
3812	-8,9231	2,8343	-10,0789	3,4041	,114	,083
4812	-8,5385	2,3218	-8,2895	2,6803	,235	,639
6031	-7,7308	2,4344	-7,1053	1,6240	,008	,148

**Note:** (1) Levene test for equality of variances, (2) t-Student test for independent samples

The best signal/noise ratio amplitude mean values were seen in the frequency range where the distortion product response amplitudes were broader and the background noise was lower.

When we compared the response amplitude mean values by the t-Student test (independent samples) in relation to gender for the control group, we observed a statistically significant difference (p>0.05) in two F2 frequencies only (1187 Hz and 1500 Hz), and males had better average values (11.44 dBSPL and 14.46 dBSPL) when compared to females (8.44 dBSPL and 10.44 dBSPL).

In regards of background noise, the mean values did not present statistically significant differences between males and females compared to the control group.

For the study group response amplitude mean values (Table 13), the t-Student test (independent samples) for gender comparison, showed a statistically significant difference in one F2 frequency only (2406 Hz); when females presented a better mean value (16.28 dBSPL) when compared to males (13.28 dBSPL).

**Table 13:** mean values, standard deviation and t-student test (independent samples) in order to compare DP mean values between the control and study groups

F2 Hz	Control		Study		p-value ^1^ F	p-value ^2^ t
	Mean value DP	Standard Deviation	Mean value DP	Standard Deviation		
593	10,7000	8,3106	10,5857	9,7987	,285	,937
750	4,4222	8,1750	5,1429	6,3504	,077	,544
937	7,1556	6,5718	7,6571	6,3426	,360	,627
1187	10,1778	6,7167	9,4429	6,7516	,931	,494
1500	12,7667	7,3577	14,0143	6,4189	,278	,263
1906	15,1778	6,7866	14,7143	6,3978	,355	,661
2406	14,4778	5,3026	14,9143	6,0090	,397	,627
3031	10,2333	4,8112	9,8429	4,6924	,653	,607
3812	10,4556	5,5347	10,5571	5,1376	,584	,906
4812	7,3333	5,8598	7,9000	5,1136	,192	,522
6031	3,8889	4,6433	3,8429	5,8697	,119	,956

**Note:** (1) Levene test for equality of variances, (2) t-Student test for independent samples.

There was no statistically significant difference among background mean values between the genders (table 14), when the t-Student test was used (independent samples).

**Table 14:** mean values, standard deviation and t-student test (independent samples) in order to compare BN mean values between the control and study groups

F2 Hz	Control		Study		p-value ^1^ F	p-value ^2^ t
	Mean value BN	Standard Deviation	Mean value BN	Standard Deviation		
593	9,7889	7,8146	8,3000	8,7499	,256	,258
750	4,0111	7,2165	1,7000	7,5572	,490	,051
937	1,4778	5,1499	,3429	5,6309	,471	,186
1187	,3000	5,3725	-2,3143	5,2765	,892	,002 (*)
1500	-2,5556	5,5244	-3,0714	5,3197	,414	,552
1906	-5,0222	4,1080	-5,2857	4,4303	,981	,698
2406	-7,8000	3,2471	-7,9714	3,2212	,837	,740
3031	-9,3333	3,9434	-9,7143	3,6679	,858	,533
3812	-9,4111	3,1226	-9,2286	3,1029	,839	,713
4812	-8,4333	2,4680	-8,4429	2,6899	,751	,981
6031	-7,4667	2,1421	-7,7429	2,7117	,034	,486

Note: (1) Levene test for equality of variances, (2) t-Student test for independent samples.

(*) Significant at 5%

According to what was presented, we can notice that while males had better distortion product mean amplitude responses in two frequencies when compared to the control group, the same thing did not happen for the study group, in which women had better distortion product amplitude mean values in one frequency only.

We can see that exposure to occupational noise during pregnancy did not affect the hearing of children born from exposed mothers. The literature is scarce in studies about newborns’ and infants’ hearing, whose mothers were exposed to occupational noise during pregnancy. Lalande et al.^27^ (1986), in their study, stated that the proportion of children with significant hearing loss in 4 KHz was 3 to 4 times greater when their mothers had been exposed to sound pressures of 85 to 95 dBSPL in comparison to lower noise doses, and also that the lower frequencies are more affected.

Ando and Hattori^23^ (1970) reported on the greater skill the child had in adapting to the growing environmental noise when their mothers were exposed to noise during pregnancy, however they did not evaluate possible hearing loss. Gerhardt and Abrams^21^ (2000) reported hearing loss in the fetuses of mothers who were exposed to noise during pregnancy. However, in our study we did not notice hearing loss in fetuses from mothers exposed to occupational noise.

## CONCLUSION

We did not notice a harmful effect on the hearing of children born from women exposed to occupational noise during pregnancy - evaluated by means of distortion product otoacoustic emissions, when compared to children born from women who were not exposed to occupational noise during pregnancy.
